# Impact of pyrrolidine-bispyrrole DNA minor groove binding agents and chirality on global proteomic profile in *Escherichia Coli*

**DOI:** 10.1186/1477-5956-11-23

**Published:** 2013-05-23

**Authors:** Ya-ting Yang, Chun-Yu Lin, Jingyueh Jeng, Chi-Wi Ong

**Affiliations:** 1 Department of Chemistry, National Sun Yat-sen University, No. 70, Lienhai Rd., Kaohsiung, 80424, Taiwan; 2 Department of Biotechnology, Chia Nan University of Pharmacy & Science, No. 60, Sec. 1, Erren Rd., Rende Dist., Tainan City, 71710, Taiwan

**Keywords:** *Escherichia coli*, Pyrrolidine-bispyrroles, Proteomics, Chirality, Matrix-assisted laser desorption/ionization (time of flight), Protein up- and down-regulation

## Abstract

**Background:**

There is great interest in the design of small molecules that selectively target minor grooves of duplex DNA for controlling specific gene expression implicated in a disease. The design of chiral small molecules for rational drug design has attracted increasing attention due to the chirality of DNA. Yet, there is limited research on the chirality effect of minor groove binders on DNA interaction, especially at the protein expression level. This paper is an attempt to illustrate that DNA binding affinity might not provide a full picture on the biological activities. Drug interacting at the genomic level can be translated to the proteomic level. Here we have illustrated that although the chiral bispyrrole-pyrrolidine-oligoamides, PySSPy and PyRSPy, showed low binding affinity to DNA, their influence at the proteomic level is significant. More importantly, the chirality also plays a role. Two-dimensional proteomic profile to identify the differentially expressed protein in *Escherichia coli* DH5α *(E coli* DH5α) were investigated.

**Results:**

*E coli* DH5α incubated with the chiral PySSPy and PyRSPy, diastereomeric at the pyrrolidine ring, showed differential expression of eighteen proteins as observed through two dimensional proteomic profiling. These eighteen proteins identified by MALDI_TOF/TOF MS include antioxidant defense, DNA protection, protein synthesis, chaperone, and stress response proteins. No statistically significant toxicity was observed at the tested drug concentrations as measured via MTT assay.

**Conclusion:**

The current results showed that the chiral PySSPy and PyRSPy impact on the proteomic profiling of *E coli* DH5α, implicating the importance of drug chirality on biological activities at the molecular level.

## Background

**Figure 1:**
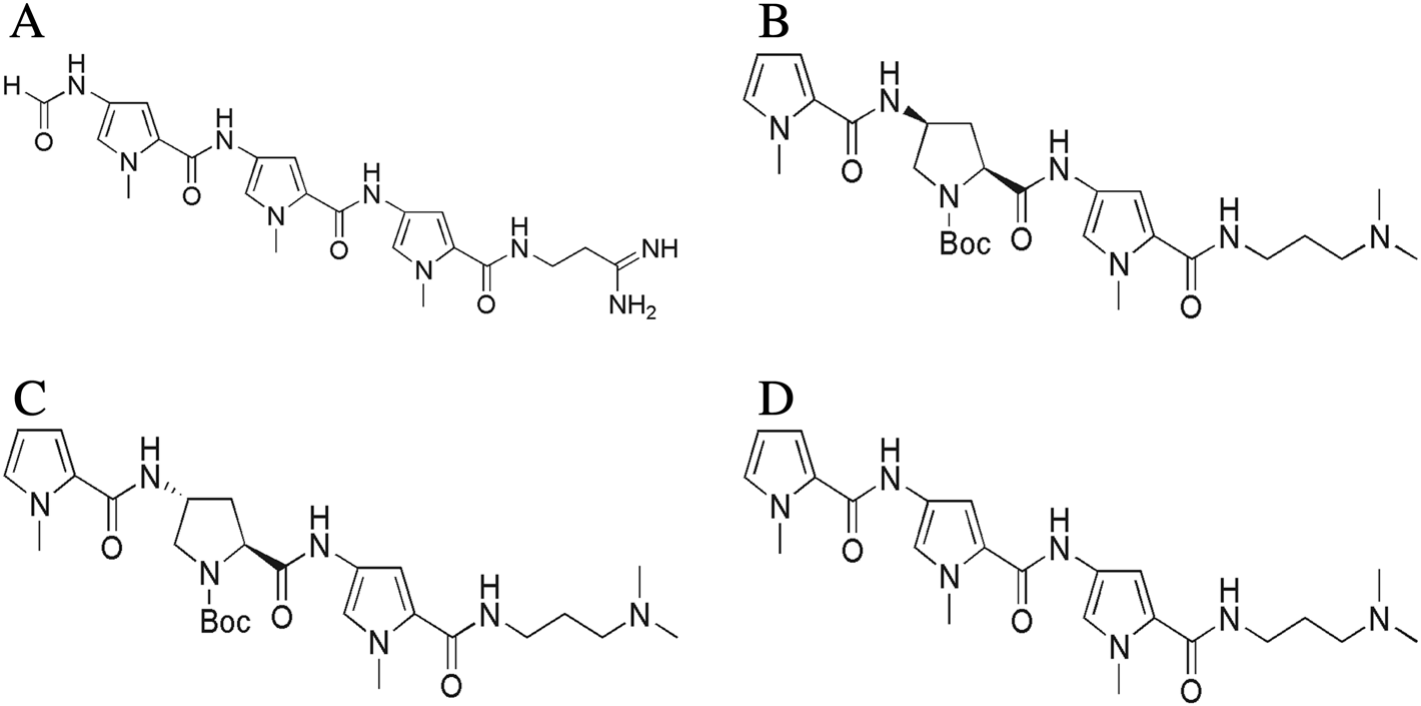
**Chemical structures of distamycin A, PySSPy, PyRSPy and 3A.** (**A**) Distamycin A. (**B**) PySSPy (**C**) PyRSPy (**D**) Tripyrrole-oligoamide (3A), a distamycin-A derived achiral compound retaining the middle pyrrole group.

Genomics has dramatically altered the way of drug discovery, and as such, small molecules with the ability to sequence-selectively recognize and discriminate between DNA have become increasingly important for the control of specific processes involved in gene expression implicated in diseases [1-5]. The minor grooves of DNA have been shown to play important roles in the regulation of gene expression and are normally unoccupied [6], hence they are considered a susceptible site of attack by small molecules less than 1000 kDa [7]. Consequently much interest has been concentrated in the design of small molecules that selectively target minor grooves of duplex DNA in order to control specific gene expression implicated in a disease [1]. There is limited research investigating the chirality effect of minor groove binders on DNA interaction. The dimerization of two N-methylpyrrole oligopeptides through the chiral linker methanodiazocin scaffold has shown that the (4R,9R)-form is better suited for interaction with calf thymus DNA than the (4S,9S)-form [8]. In addition, Herman *et al.* (1998) has shown that the enantiomer derived from two distamycin A-derived polyamides linked by either (R)-2,4-diaminobutyric acid showed enhanced binding affinity towards DNA when compared to the (S)-2,4-diaminobutyric acid-enantiomer [9]. Our laboratory has recently designed and synthesized two distamycin A-derived diastereoisomers, depicted as PySSPy and PyRSPy [10]. These two diastereoisomers have their middle pyrrole group replaced with a pyrrolidine group and the terminal amide group removed (Figure 1).

Genome research has led to medical advances against diseases [11,12]. However, human complexity cannot be entirely explained by its genomics, but by the way their gene products interact. To further complicate the matter, genome is a static process while proteome is a dynamic process. In other words, one gene can encode for a number of proteins. While most drugs target proteins, disease profiling is at present dominated by DNA microarrays [13-15]. As such, there may not be a good correlation between gene expression and protein expression in most disease processes, and treatments can be manifested at the protein level. Thus, it is believed that gene-based expression analysis alone is inadequate for drug discovery.

In the past, protein expression has been analyzed through mRNA studies. However, it was later shown that mRNA content does not correlate with protein content as mRNA is not always translated into protein [16-19]. On the other hand, proteomics is a systematic analysis that measures protein expression directly and not via gene expression, yet serving as a complementary approach to genomics. 2D gel electrophoresis is still the most useful way to separate proteins in complex samples in proteomics profiling and allows simultaneous analyses of vast amount of protein data, making it suitable for comparative analysis of a reference cell protein profile with a profile after drug treatment in the search of new drug or drug target. At present there are minimal researches on the consequences of DNA minor groove binding agents on the proteomic profile in cells.

*Escherichia coli* (*E. coli*) are one of the best-characterized prokaryotes. Since the completion of the *E. coli* genome-sequencing project [20], this organism has been studied on the genome wide scale in terms of its transcriptome, proteome, interactome, metabolome, and physiome [21-25]. The uses of proteomics technologies on *E coli* have generated unprecedentedly large amounts of proteome data for *E coli*[25-27]. This extensive *E coli* proteome database has therefore made *E coli* an ideal model organism for a large-scale comparison of protein expression levels under particular chemical and physical stresses [26]. Our initiative is to examine whether proteomics is a more realistic end point for drug action, and at the same time the chirality effects on protein expression. To achieve this goal, we have synthesized chiral compounds PySSPy and PyRSPy and performed the proteomic profiling analyses on an *E. Coli* DH5α strain by performing 2D gel electrophoresis coupled with mass spectrophotometry to determine the expressional changes on different proteins. This method allows us to carry out a simultaneous search on a vast number of proteins that may have expressional changes. We have also examined the protein expressional changes of *E. Coli DH5α* in response to tripyrrole-oligoamide (3A). This compound is also a distamycin A-derived compound, which retains the middle pyrrole group that is achiral. The proteomic profiling of the achiral 3A is examined to confirm the influence of chirality. Although there are numerous differentially expressed proteins, in this work we have identified the most significant eighteen proteins that have either been up- or down-regulated, and the chirality of the compound was found to play an important role.

## Results

### Susceptibility of *E coli* DH5α to bispyrrole-pyrrolidines

To examine the susceptibility of *Escherichia coli* DH5α (*E coli* DH5α) towards PySSPy, *E coli* DH5α treated with variable PySSPy concentrations were examined for their cell growth via OD_600_ analyses (Additional file 1). The growth curve of the non-treated cells exhibited a 6-hour lag phase, followed by a 6-hour exponential phase and subsequently a stationery phase. At 9-hr incubation, *E coli* DH5α treated with 100, 150, 200 and 300 μM PySSPy showed approximately 8 (p < 0.01), 10 (p < 0.001), 12 (p < 0.001) and 20% (p < 0.001) decreases in cell density, respectively. However, the growth curves of the PySSPy treated cells eventually reached the same plateaus as that of the control cells. The results indicate the non-toxic nature of PySSPy on *E coli* DH5α. A concentration of 300 μM was subsequently chosen for the rest of the study to maximize the possible effects of PySSPy on *E coli* DH5α proteome.

**Figure 2:**
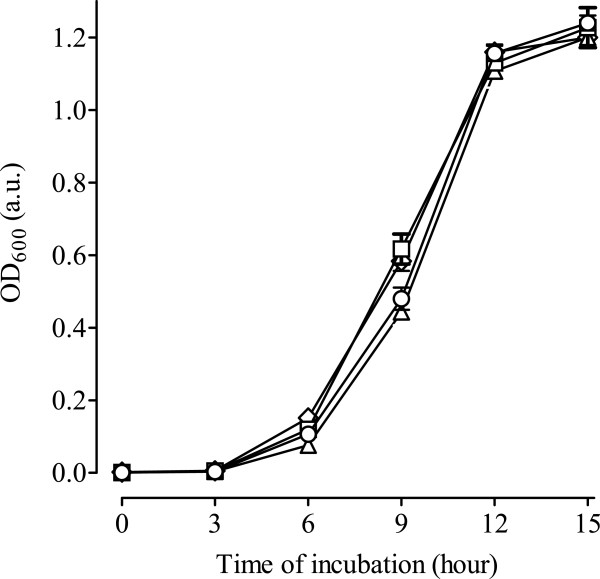
***E. coli *DH5α growth after treatment with PySSPy, PyRSPy and 3A.***E. coli* DH5α in LB broth were treated with 0 (square), or 300 μM PySSPy (triangle), PyRSPy (circle), and 3A (diamond) at 37°C for 15 hours. Cell density was determined at time intervals through OD_600_ measurement. Each data point was subtracted from blank controls. Each data point was the mean result of triplicate values. Standard deviations were calculated for each data point and presented as error bars.

The cytotoxicity of PySSPy, PyRSPy and 3A was subsequently compared (Figure 2). Exposure of *E coli* DH5α to either 300 μM PySSPy or PyRSPy resulted in approximately 20% decreases in cell viability at 9-hr time period (p < 0.001), after which the cell growth curves became comparable to that of the control (p > 0.05). Exposure of *E coli* DH5α to 300 μM 3A resulted in no statistically significant decreases in cell density over time. Overall, these results suggest that PySSPy and PyRSPy have limited cytotoxicity effects on *E coli* DH5α even at a high concentration of 300 μM.

### Proteomic analyses

The lack of cytotoxicity observed in PySSPy- and PyRSPy- and 3A-treated *E. coli* DH5α allows the further study of their influence at the molecular level associated with the global protein expression. Differential protein expression in *E. coli* DH5α cells following exposure to 300 *μ*M PySSPy, PyRSPy and 3A for 9 hours were examined using a comparative proteomic analysis. Protein spots resulted from the PySSPy-, PyRSPy- and 3A-treated 2-DE gels were compared with the corresponding protein spots from the control gel. The proteomic profiles of total proteins from the control and treated *E. coli* DH5α from three independent experiments were shown in Additional files 2, 3, 4 to 5.

**Figure 3:**
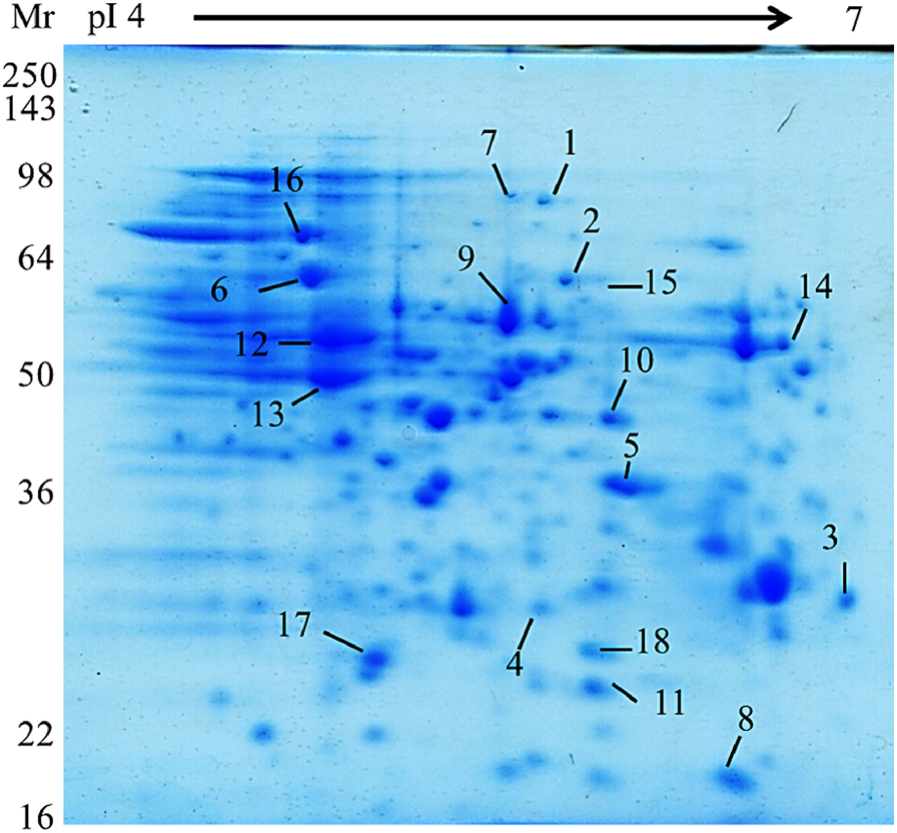
**Two-dimensional proteomic map of soluble proteins in *E. coli *DH5α.***E. coli* DH5α were treated in the absence of any distamycin A derivatives at 37°C for 10 hours, followed by cell protein solubilization via high intensity ultrasonication in rehydration buffer. The soluble protein (240 μg) was separated on an immobilized pH gradient gel strip (pH 4-7) and 12% SDS-PAGE gel. Proteins were visualized by staining with commercial SimplyBlue SafeStain. The numbers correspond to the numbers listed in Figure 4 and Table 1.

**Figure 4:**
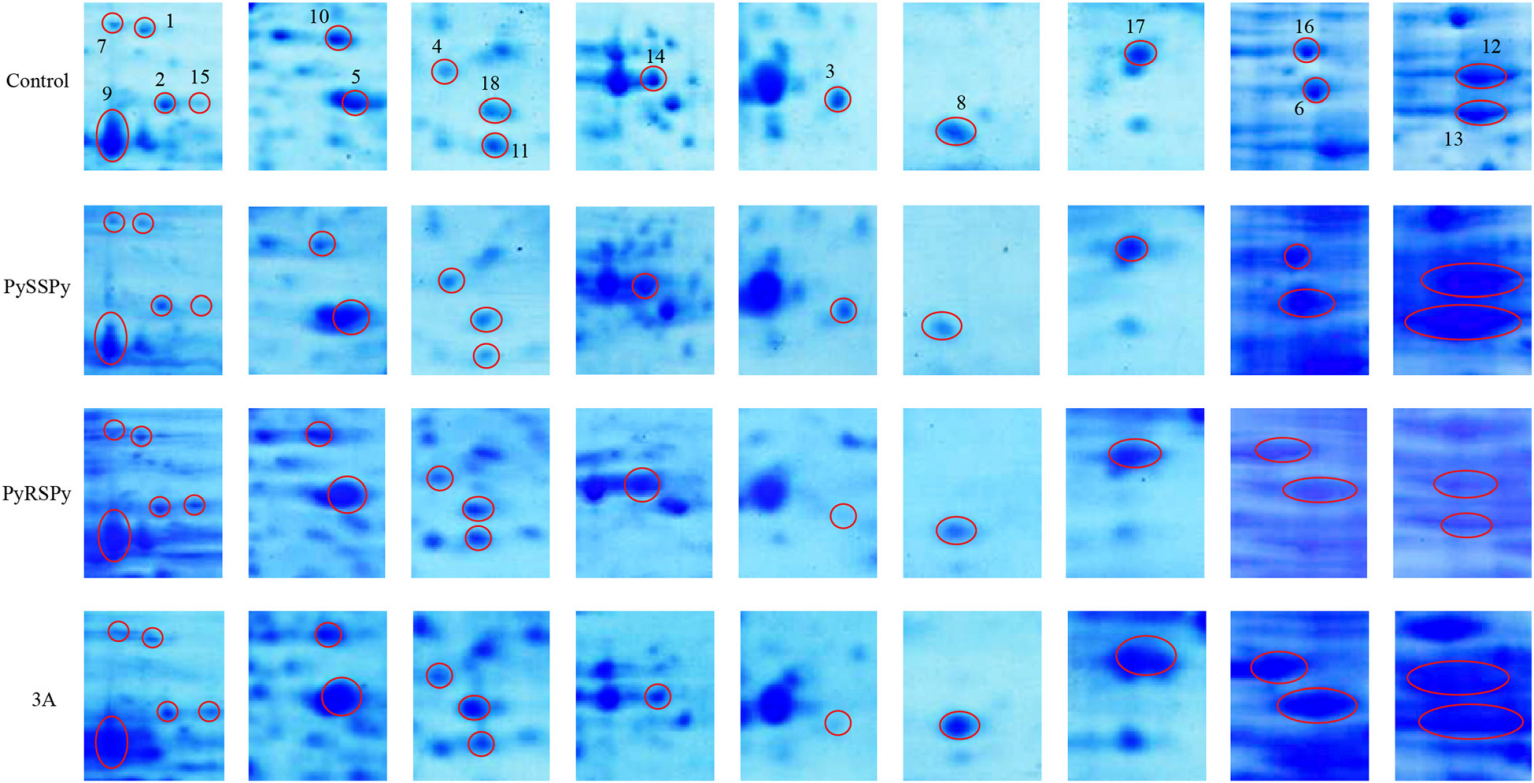
**The expression of the proteins identified in *E. coli *DH5α after treatment with distamycin-A derivatives.***E. coli* DH5α were treated in the absence (control) or presence of 300 μM PySSPy, 300 μM PyRSPy or 300 μM 3A at 37°C for 10 hours, followed by cell protein solubilization and protein separation using an immobilized pH gradient gel strip (pH 4-7) and 12% SDS-PAGE gel. The gels were visualized using commercial SimplyBlue SafeStain. The resulting proteomic maps were shown in enlarged sections for comparison with the non-treated control. The numbers represent the proteins that show either up- or down-regulation in *E. coli* DH5α in response to PySSPy, PyRSPy and 3A, compared with the non-treated control. These numbers correspond to the numbers listed in Figure 3 and Table 1.

The representative proteomic profile of total proteins from the control *E. coli* DH5α showed that proteins were widely distributed across the pI range (4-7) between the molecular weight 16 and 143 kDa (Figure 3). After comparison with the control, some protein spots from the PySSPy-, PyRSPy- and 3A-treated gels with significant expressional change were excised from the gels (Figure 4) and identified by MALDI-TOF mass spectrometry using peptide mass fingerprint searches (Table 1). Eighteen protein spots in total were successfully identified with significant scores and high sequence coverage. The abundance of the remaining protein spots were either too low to be detected or the scores did not reach statistical significance. The data suggests that PySSPy. PyRSPy and 3A at a non-cytotoxic concentration can still induce alteration in protein expression.

**Table 1:** **Identification of differentially expressed proteins of *E coli *DH5α treated with PySSPy, PyRSPy and 3A**

**No.**	**ICBI accession No.**	**Protein identified**	**Calculated pI/MW (kDa)**	**Matched (Measured) peptide**	**Sequence coverage (%)**	**Relative intensities**^**a**^		
						**PySSPy**	**PyRSPy**	**3A**
1	GLYA_EC024	Serine hydroxymethyl-transferase	6.03/94	9 (22)	29	-25 ± 04	-2.1 ± 0.2	-1.4 ± 0.1
2	IR3Z2_ECOUT	Glycerol kinase	5.97/60	15 (30)	33	-2.3±0.4	-1.6±0.05	-1.7±0.1
3	DHSB_ECQU	Succinate dehydrogenase iron-sulfur subunit	6.327	10 (21)	47	-2.4 ± 0.1	-3.5 ± 0.3	-3.4 ± 0.1
4	KDUD_ECOLI	2-dehydro-3-deoxy-D-gluconate 5-dehyrixenase	5.24/27	8 (18)	40	+1.7 ± 0.1	+2.0 ± 0.2	+1.9 ± 0.2
5	IvEH_ECO24	Malate dehydrogenase	5.61/32	11 (22)	49	+2.3 ± 0.2	+3.1 ± 0.05	+1.9 ± 0.06
6	CH6O1_ECOK1	60 kDa chaperonin 1	4.85157	10 (26)	33	+2.4 ± 0.1	+2.5 ± 0.2	+3.1 ± 0.4
7	MAO2_ECOLI	NADP-dependent malic enzyme	5,3494	11 (13)	14	-21 ± 0.5	-2.1±0.3	NS
8	DPS_EC024	DNA protection during starvation protein	5.72/19	9 (1)	58	-2.3 ± 0.1	-2.8 ± 0.6	NS
9	PCKA_EC024	Phosphoenolpyruvate carboxykinase	5.46/60	14 (27)	35	-24 ± 0.2	NS	NS
10	GCST_EC024	Aminomethyltransferase	5.36/40	9 (17)	35	-2.1 ± 0.3	NS	NS
11	SODF_ECO57	superoxide dismutase [Fe]	5.58/53	15 (16)	37	NS	+1.9 ± 0.009	NS
12	ATPB_EC024	ATP synthase subunit beta	4.9150	16 (50)	50	+3.0±0.2	NS	+2.6 ± 0.2
13	EFTIJ1_EC024	Elongation factor Tu 1	53143	17 (44)	53	+3.3 ± 0.2	NS	+3.1 ± 0.1
14	TNAA_EC024	Tryptophanase	5.88/53	15 (16)	37	NS	+1.9 ± 0.009	NS
15	PUR9_ECO5S	Bifunctional purine biosynthesis protein purH	5.53/58	11 (14)	31	NS	+1.3 ± 0.2	+1.6 ± 0.06
16	DNAK_ECO24	Chaperone protein dnaK	4.83/69	16 (28)	28	NS	+3.0 ± 0.1	+2.1 ± 0.2
17	AHPC_ECO57	Alkyl hydroperoxide reductase subunit C	5.0/21	6 (11)	43	NS	+2.6 ± 0.3	+2.5 ± 0.4
18	ALKH_ECO57	KHG/KDPG aldolase	5.57/22	7 (20)	46	NS	+1.8 ± 0.2	+2.1 ± 0.3

^a^ “-” indicates down-regulation and “+” indicates up-regulation. The numerical values indicate the relative intensities of protein spots of interest in comparison with the corresponding control proteins, and they are presented as mean ± standard deviations of at least 2 independent proteomic gels. “NS” indicates the intensity changes are statistically non-significant in comparison to the corresponding control proteins.

The 18 proteins in *E coli* DH5α identified that showed differential expression in response to either PySSPy or PyRSPy or both are listed in Table 1. Both PySSPy and PyRSPy induced significant down-regulation of serine hydroxymethyltransferase, glycerol kinase, succinate dehydrogenase iron-sulfur subunit, NADP-dependent malic enzyme and DNA protection during starvation protein. Malate dehydrogenase, 2-dehydro-3-deoxy-D-gluconate 5 dehydrogenase and 60 kDa chaperonin 1 were significantly up-regulated by both PySSPy and PyRSPy. Interestingly, selective proteins were differentially expressed in response to either PySSPy or PyRSPy alone. PySSPy alone significantly down-regulated phosphoenolpyruvate carboxykinase, aminomethyltransferase and superoxide dismutase [Fe], and up-regulated ATP synthase subunit beta and elongation factor Tu 1. PyRSPy alone significantly up-regulated tryptophanase, bifunctional purine biosynthesis protein purH, chaperone protein dnaK, alkyl hydroperoxide reductase subunit C and KHG/KDPG aldolase. In addition, the control 3A alone did not induce differential protein expression. The results suggest these two diastereoisomers differing only in their chiral center show protein selectivity, whereas the chiral 3A is less selective in protein expression regulation.

The proteins identified can be classified into several groups using the UniProtKB database and the KEGG database based on their molecular functions, including transferases, oxidoreductases, lyases, hydrolases, elongation factor, and chaperone. These proteins are involved in metabolism (serine hydroxymethyltransferase, NADP-dependent malic enzyme, glycerol kinase, phosphoenolpyruvate carboxykinase, aminomethyltransferase, 2-dehydro-3 deoxy-D-gluconate 5-dehydrogenase, succinate dehydrogenase iron-sulfur subunit, ATP synthase subunit beta, bifunctional purine biosynthesis protein purH, tryptophanase, KHG/KDPG aldolase and malate dehydrogenase), antioxidant defense (superoxide dismutase [Fe], DNA protection during starvation protein and alkyl hydroperoxide reductase subunit C), protein synthesis (elongation factor Tu 1), DNA replication (chaperone protein dnaK) and stress response (60 kDa chaperonin 1), rather than taking part in a wide variety of biological processes.

## Discussion

Selective and cytostatic anticancer agents may not express their biological activities through cell death or tumor shrinking, but on the tuning of the biochemical activities of protein to effect survival. The synthesized PySSPy, PyRSPy and 3A showed weak binding to calf thymus DNA [10] and low cytotoxicity (Figure 2). Even so, the current study using 2- dimensional gel electrophoresis coupled with mass spectrophotometry [28-31] has allow us to identified 18 responsive proteins involved in different biological processes to be up- or down regulated by these compounds (Figure 4 and Table 1). This illustrate that proteomics may be a more important to fully expressed the interaction of drugs at the cellular level. Here, PySSPy and PyRSPy were shown to selectively affect several proteins, and these results are promising.

The achiral compound 3A was found to be less selective in influencing particular protein expression. Its impacts on protein expression seemed to be more random as compared to those of PySSPy and PyRSPy, implying the importance of chirality in regulating selective protein expression. The 18 proteins with altered expression level in PySSPy- and PyRSPy-treated *E coli* DH5α can be categorized into a broad range of different functional classes, including metabolism, cellular defenses, DNA replication, stress responses, and protein synthesis. The wide range of different proteins involved suggested that PySSPy and PyRSPy can induce profound biological responses in *E coli* DH5α.

Among the 18 proteins, PySSPy alone has shown to down-regulation of 3 proteins and up regulation of 2 proteins, whereas PyRSPy alone up-regulated 5 proteins (Table 1). This result suggests that PySSPy and PyRSPy that differ only in configuration of one stereogenic center can alter the expression of selective genes. This indicates that these chiral compounds may exert differential cellular effects. The present preliminary data illustrate the importance of coupling proteomics studies with genomic studies to obtain a clearer picture for the biological activities of drugs. Of interest is the down-regulation of phosphoenolpyruvate carboxykinase by PySSPy because this protein is involved in gluconeogenesis and catalyzes the conversion of oxaloacetate to phosphoenolpyruvate in humans. This metabolite can be converted to pyruvate in the glycolytic pathway or to glucose in the gluconeogenesis pathway.

Another noteworthy finding in the present study is the up-regulation of chaperone protein dnaK (DnaK) after PyRSPy treatment (Table 1). DnaK is a chaperone protein in bacteria belonging to the heat shock 70 kDa (Hsp70) chaperone protein family. When bound to ADP, DnaK binds to exposed hydrophobic residues of unfolded or partially mis-folded proteins, stabilizing them and giving them time to fold correctly [32]. DnaK is also involved in stabilizing precursor proteins, assisting the translocation of newly synthesized proteins, and protecting cells against cellular stress [33]. For many years Hsp70 in humans has been implied to play a neuroprotective role in some neuron degenerative diseases [34]. Hsp70 levels have been shown to decrease along with disease progression in the Huntington’s diseased mouse brain, implicating its role in preventing the pathogenesis of the disease [35]. Furthermore, Hsp70 overexpression has been shown to reduce *α*-Syn protein aggregate formation and toxicity in a mouse model of Parkinson’s disease [36], and has caused mice to be more resistant to neurodegeneration following brain ischemia [37]. Hence, it is important to examine the effects of PySSPy and PyRSPy on the expressional changes of phosphoenol-pyruvate carboxykinase and DnaK, respectively, and the toxicity effects of these two compounds using a human cell line system to address their possible clinical importance.

## Conclusion

We have shown for the first time through a proteomic approach that distamycin A-derived chiral compounds selectively regulate the expression of proteins at the molecular level. These compounds’ non-cytotoxic nature are implicative of their potential in altering a particular cellular pathway without inducing cellular toxicity in host cells, hence offering the prospect of new drug development. The future studies are aimed at investigating whether these compounds exerted the same effects on human cells as on bacterial cells. Furthermore, the significance of these compounds in altering a particular cellular pathway and treating particular diseases remain to be determined.

## Methods

### Chemicals and reagents

Tris-HCl, tris-base, phosphate buffer saline (PBS), 3-[(3-cholamido-propyl)di-methylammonio]-1-propanesulfonate (CHAPS), iodoacetamide and acrylamide (40%) were purchased from Amresco (OH, USA). Urea, glycerol, ammonium persulfate, and ammonium bicarbonate were purchased from Showa Chemicals Inc. Bradford protein assay kit and dithiothreitol (DTT) were obtained from GMbiolab Co. Ltd. Formic acid, trifluoroacetic acid and N,N,N',N'-Tetramethyl-1-,2-diamino-methane were purchased from Sigma-Aldrich, Co. Acetonitrile, thiourea and sodium dodecyl sulfate (SDS) were obtained from Merck. Pre stained protein marker and SimplyBlue SafeStain were purchased from Invitrogen (NY, USA), and α-cyano-4-hydroxy-cinnamic acid from Tokyo Kasei Kogyo Co., Ltd (TCI). Immobilline Dry Strips (pH 4-7, 7 cm, linear gradient) and immobilized pH gradient (IPG) buffer (pH 4-7) were purchased from GE healthcare.

### Cell culture and treatment

A single colony of *Escherichia coli* DH5α (*E coli* DH5α) removed from agar containing Lysogeny broth (USB Products) was suspended in each of the 100 ml of lysogeny broth media and grown for 24 hours by shaking at 160 rpm at 30°C. Subsequently, the cells were transferred into fresh lysogeny broth media (0.03% (v/v)) for a further 12 hours in the absence or presence of PySSPy, PyRSPy and 3A.

### Cell growth determination

*E coli* DH5α were assessed for their cell growth by measuring their turbidity at 600 nm (OD_600_) using an ELISA plate reader (Thermo Multiskan Ascent). Triplicate samples from each treatment were obtained for the determination of mean values and standard deviations.

### Sample preparation for isoelectric focusing (IEF)

Cells collected at the end of the incubation period were washed with phosphate buffer saline (PBS) and the final time with nanopure water at 9000 rpm at 15°C. The cell pellet was subsequently lysed by re-suspension in 0.5 ml rehydration buffer (7 M urea, 2 M thiourea and 4% (w/v) CHAPS) and ultrasonication (Elmasonic E70H) for a total of 1 minute on ice. The supernatant, after centrifugation of the lysate at 12 000 rpm at 4°C, was measured for protein content using the Bradford protein determination kit and stored at -20°C.

### Isoelectric focusing and second dimension SDS-PAGE gel electrophoresis

Protein samples (240 μg) were prepared by precipitation of the protein supernatant in 70% acetonitrile on ice for 20 minutes. The protein pellet was then dissolved in 120 μl of rehydration buffer (7 M urea, 2 M thiourea and 4% (w/v) CHAPS), 50 mM dithiothreitol (DTT) and 2% IPG buffer (pH 4-7).

After rehydrating the 7-cm IPG strips (pH 4–7) with the samples for 15 hours, isoelectric focusing (IEF) was conducted on these IPG strips by using a Protean IEF Cell (Bio-Rad). These samples required 100–24 000 voltage-hours for optimum focusing. After focusing, a conditioning step was conducted by treating the strips in equilibration buffer I (6 M urea, 0.375 M Tris-HCl, pH 8.8, 2% SDS (w/v), 20% glycerol (w/v) and 130 mM DTT) for 30 minutes and equilibration buffer II (6 M urea, 0.375 M Tris-HCl, pH 8.8, 2% SDS (w/v), 20% glycerol (w/v) and 135 mM iodoacetamide) for another 30 minutes.

The second dimension (2D) SDS-PAGE gel was run by using 12% resolving gels composed of 12% acrylamide, 0.375 M Tris-HCl, pH8.8, 0.1% SDS, 0.1% ammonium persulfate, 0.08% (v/v) N,N,N',N'-Tetramethyl-1-,2-diamino-methane, and 0.5% stacking gel (0.5% (w/v) agarose and 0.002% (w/v) bromophenol blue). The gels were stained with SimplyBlue SafeStain and directly scanned using a Microter 4100 scanner. The corresponding protein spots of interest from each different gel image were analyzed for their intensities using the ImageJ software (version 1.46), available on http://rsbweb.nih.gov/ij/download.html. The protein intensities were normalized by dividing the intensities of the protein spots of interest by the intensity of a chosen protein spot in the same gel, whose intensity is considered not significantly changed across all the control and treated gels. The relative intensities of protein spots of interest were calculated by dividing the intensities of protein spots of interest by the intensities of the corresponding protein spots in the controls. The mean fold changes and the standard deviations in Table 1 were obtained from triplicate gel samples.

### In-gel digestion, MALDI-MS analyses, and protein identification

The procedure for In-gel digestion was described by the Biological Mass Spectrometry Laboratory, the Ontario Wide Protein Identification Facility. Please refer to the website http://www.biochem.uwo.ca/wits/bmsl/index.html. Briefly, protein spots were excised and incubated with 40 μl of 50% acetonitrile in nanopure water for 15 minutes, followed by incubating the gel pieces with 100 mM ammonium bicarbonate and acetonitrile in 1:1 ratio for 15 minutes. The gel pieces were subsequently dried in a speedvac and incubated with 40 μl of 50 mM DTT/100 mM ammonium bicarbonate at 56°C for 45 minutes. The gel pieces, after removal of the supernatant, were incubated with 40 μl of 200 mM iodoacetamide/100 mM ammonium bicarbonate at room temperature for 45 min in the dark. After discarding the supernatant at the end of the incubation, the gel pieces were treated with 40 μl of 5 ng/μl sequencing grade modified trypsin (Promega, Madison, WI) in 25 mM ammonium bicarbonate and the mixtures were incubated overnight at 37°C. The supernatants from the trypsin-digested mixtures were collected in separate tubes, and peptides were extracted twice by treating the gel pieces with 30 μl of acetonitrile/5% formic acid in 1:1 ratio. The extracts were completely dried using a Speed Vac.

The dried peptides were dissolved in 5 μl of 0.1% formic acid. One μl of peptide samples were mixed with 1 μl of matrix solution (30 mg/ml α-cyano-4- hydroxycinnamic in 70% acetonitrile/0.1% trifluoroacetic acid) onto a matrix-assisted laser desorption/ionization (MALDI) plate, and allowed to air-dry. Spectra were acquired using Bruker Daltonics Autoflex III/Autoflex III TOF/TOF (serial number 238.120.00103). Peptide calibration for a mass range of m/z 500 to m/z 2500 was performed using α-cyano-4-hydroxycinnamic acid (α-CHCA), angiotensin I, and adrenocorticotropic hormone (ACTH). The peak lists obtained were searched using Mascot search engine, and compared against non-redundant NCBI protein database. The following parameters were used: maximum allowed missed cleavages by trypsin was 1; fixed amino acid modification as carbamidomethyl; *Escherichia coli* was chosen for the taxonomy; the peptide tolerance for spectra obtained from MALDI was ± 0.5Da. Only protein samples with MOWSE scores above the significant threshold level (p ≤ 0.05) as determined by MASCOT were listed in Table 1. The proteins identified were classified into different molecular functions, the elemental activities of a gene product at molecular level, using the Uni Protein Knowledgebase (UniProtKB) database available on http://www.uniprot.org/.

### Statistical analysis

Data was plotted and statistically analyzed using the Prism software program (GraphPad Software, USA). Significance was confirmed by a one-way analysis of variance [8] and Tukey’s multiple comparison test. Significance levels are indicated in the following manner: * p ≤ 0.05; ** p ≤ 0.01; *** p≤ 0.001. The means and standard deviations shown within each experiment were calculated from triplicate samples.

## Abbreviations

2-DE: Two-dimensional gel electrophoresis; CHAPS: 3-[(3-cholamido-propyl)dimethyl-ammonio]-1-propanesulfonate; DTT: Dithiothreitol; E coli: *Escherichia coli*; Hsp: Heat shock; IPG: Immobilized pH gradient; IEF: Isoelectric focusing; LB: Lysogeny broth; MALDI-TOF: Matrix-assisted laser desorption/ionization-time of flight; PAGE: Polyacrylamide gel electrophoresis; PBS: Phosphate buffer saline; SDS: Sodium dodecyl sulfate.

## Competing interests

The authors declare that they have no competing interests.

## Authors’ contributions

YTY, CWO and JYJ designed the study. YTY and CYL carried out the experiments. CYL designed and synthesized the compounds. YTY wrote the manuscript, and CWO and JYJ corrected the manuscript. All authors read and approved the final manuscript.

## Supplementary Material

Additional file 1 ***E. coli *DH5α cell growth after treatment with the distamycin A derivative PySSPy.***E. coli* DH5α in LB broth were treated with 0 (dark square), 50 (inverted white triangle), 100 (white triangle), 150 (white circle), 200 (white diamond), 300 (white square) μM PySSPy at 37°C for 15 hours. Cell density was determined at time intervals through OD 600 measurement. Each data point was subtracted from blank controls. Each data point was the mean result of triplicate values. Standard deviations were calculated for each data point and presented as error bars. Click here for file

Additional file 2 **The proteomic gel images of *E. coil *DH5α control.***E. coil* DH5α were treated in the absence of 300 μM PySSPy. 300 μM PyRSPy or 300 μM 3A at 37°C for 10 hours, followed by 2D gel electrophoresis. The gels were visualized using commercial SimplyBlue SafeStain. The gels images **A)**, **B)** and **C)** were obtained from three independent experiments. Click here for file

Additional file 3 **The proteomic gel images of *E. coil *DH5α treated with PySSPy.***E coli* DH5α were treated with 300 μM PySSPy at 37°C for 10 hours, followed by 2D gel electrophoresis. The gels were visualized using commercial SimplyBlue SafeStain. The gels images **A)**, **B)** and **C)** were obtained from three independent experiments. Click here for file

Additional file 4 **The proteomic gel images *of E. coil *DH5α treated with PyRSPy.***E. coli* DH5α were treated with 300 μM PyRSPy at 37°C for 10 hours, followed by 2D gel electrophoresis. The gels were visualized using commercial SimplyBlue SafeStain. The gels images **A)**, **B)** and **C)** were obtained from three independent experiments. Click here for file

Additional file 5 **The proteomic gel images of *E. coil *D115u treated with 3A**. *E. coil* DH5α were treated with 300 μM 3A at 37°C for 10 hours, followed by 2D gel electrophoresis. The gels were visualized using commercial SimplyBlue SafeStain. The gels images **A)**, **B)** and **C)** were obtained from three independent experiments. Click here for file
