# Supplementary material for: Impact of pyrrolidine-bispyrrole DNA minor groove binding agents and chirality on global proteomic profile in Escherichia Coli
Source: Proteome Sci. 2013 May 23;11:23. doi: 10.1186/1477-5956-11-23 (PMC3669006; doi:10.1186/1477-5956-11-23)
Supplement: Additional file 1 — E. coli DH5α cell growth after treatment with the distamycin A derivative PySSPy.E. coli DH5α in LB broth were treated with 0 (dark square), 50 (inverted white triangle), 100 (white triangle), 150 (white circle), 200 (white diamond), 300 (white square) μM PySSPy at 37°C for 15 hours. Cell density was determined at time intervals through OD 600 measurement. Each data point was subtracted from blank controls. Each data point was the mean result of triplicate values. Standard deviations were calculated for each data point and presented as error bars. [file 1477-5956-11-23-S1.pdf]

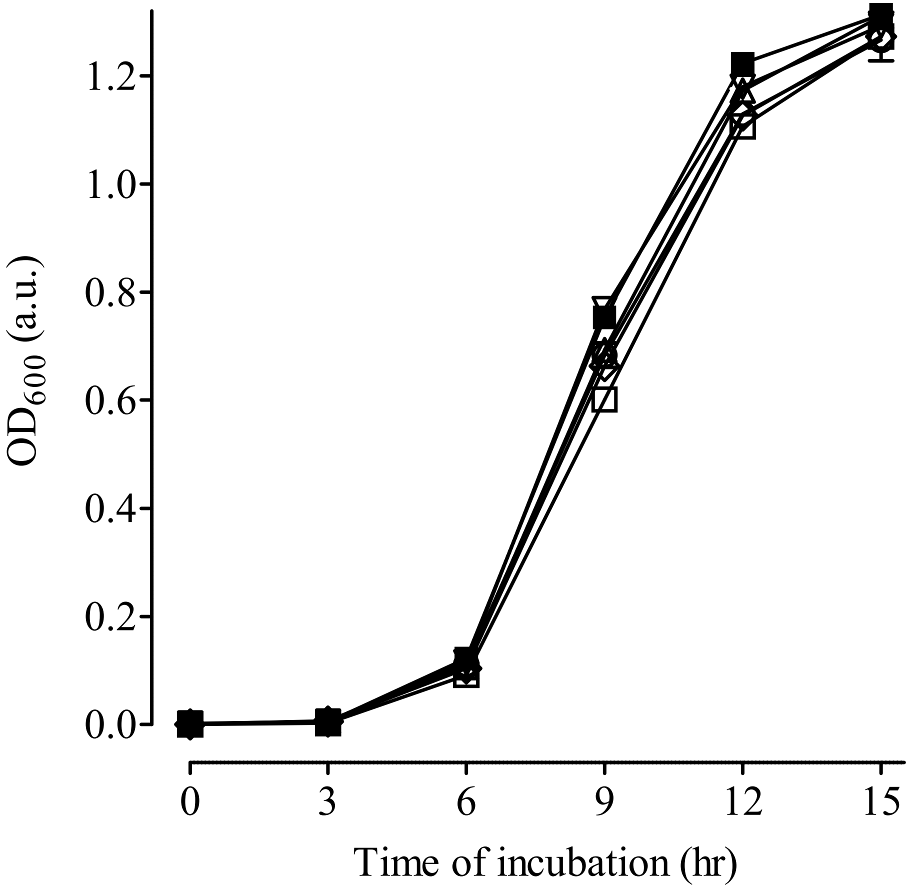

Additional file 1 ***E. coli DH5α*** **cell growth after treatment with the distamycin A derivative PySSPy.** *E. coli DH5α* in LB broth were treated with 0 (dark square), 50 (inverted white triangle), 100 (white triangle), 150 (white circle), 200 (white diamond), 300 (white square) μM PySSPy at 37℃ for 15 hours. Cell density was determined at time intervals through OD_{600} measurement. Each data point was subtracted from blank controls . Each data point was the mean result of triplicate values. Standard deviations were calculated for each data point and presented as error bars.
