# Supplementary figures and images for: Impact of pyrrolidine-bispyrrole DNA minor groove binding agents and chirality on global proteomic profile in Escherichia Coli
Source: Proteome Sci. 2013 May 23;11:23. doi: 10.1186/1477-5956-11-23 (PMC3669006; doi:10.1186/1477-5956-11-23)

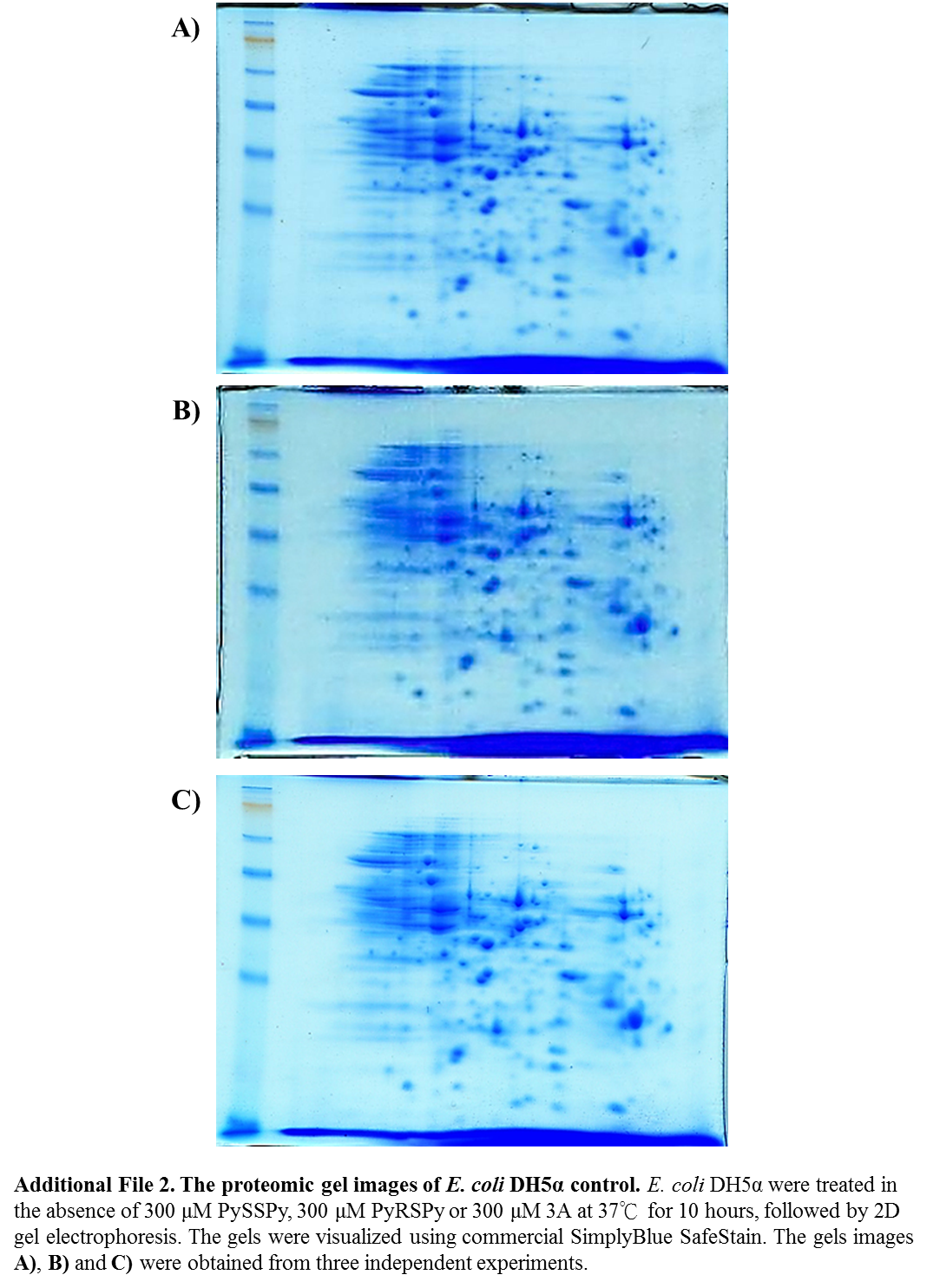

Supplement: Additional file 2 — The proteomic gel images of E. coil DH5α control.E. coil DH5α were treated in the absence of 300 μM PySSPy. 300 μM PyRSPy or 300 μM 3A at 37°C for 10 hours, followed by 2D gel electrophoresis. The gels were visualized using commercial SimplyBlue SafeStain. The gels images A), B) and C) were obtained from three independent experiments. [file 1477-5956-11-23-S2.tiff]

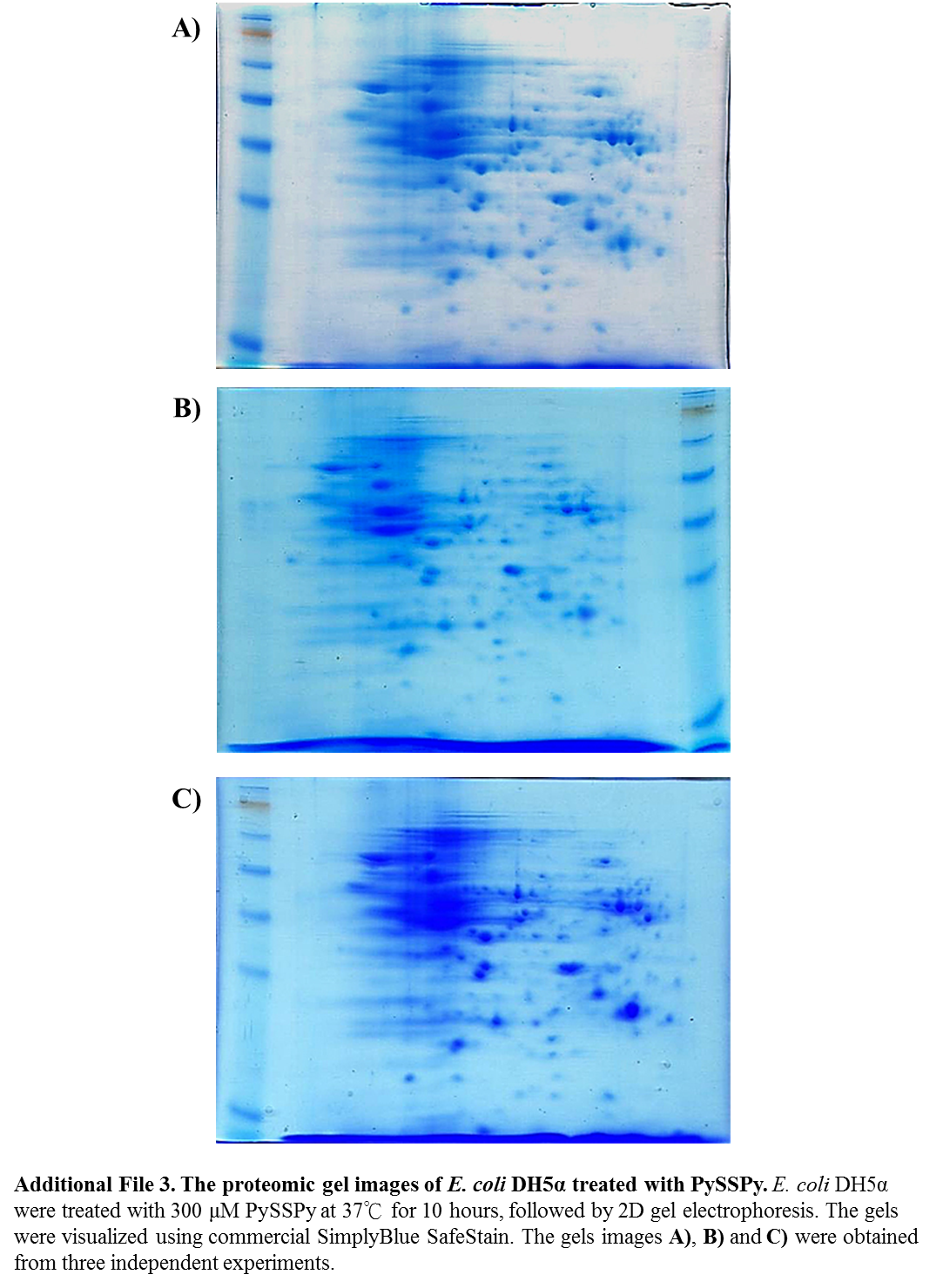

Supplement: Additional file 3 — The proteomic gel images of E. coil DH5α treated with PySSPy.E coli DH5α were treated with 300 μM PySSPy at 37°C for 10 hours, followed by 2D gel electrophoresis. The gels were visualized using commercial SimplyBlue SafeStain. The gels images A), B) and C) were obtained from three independent experiments. [file 1477-5956-11-23-S3.tiff]

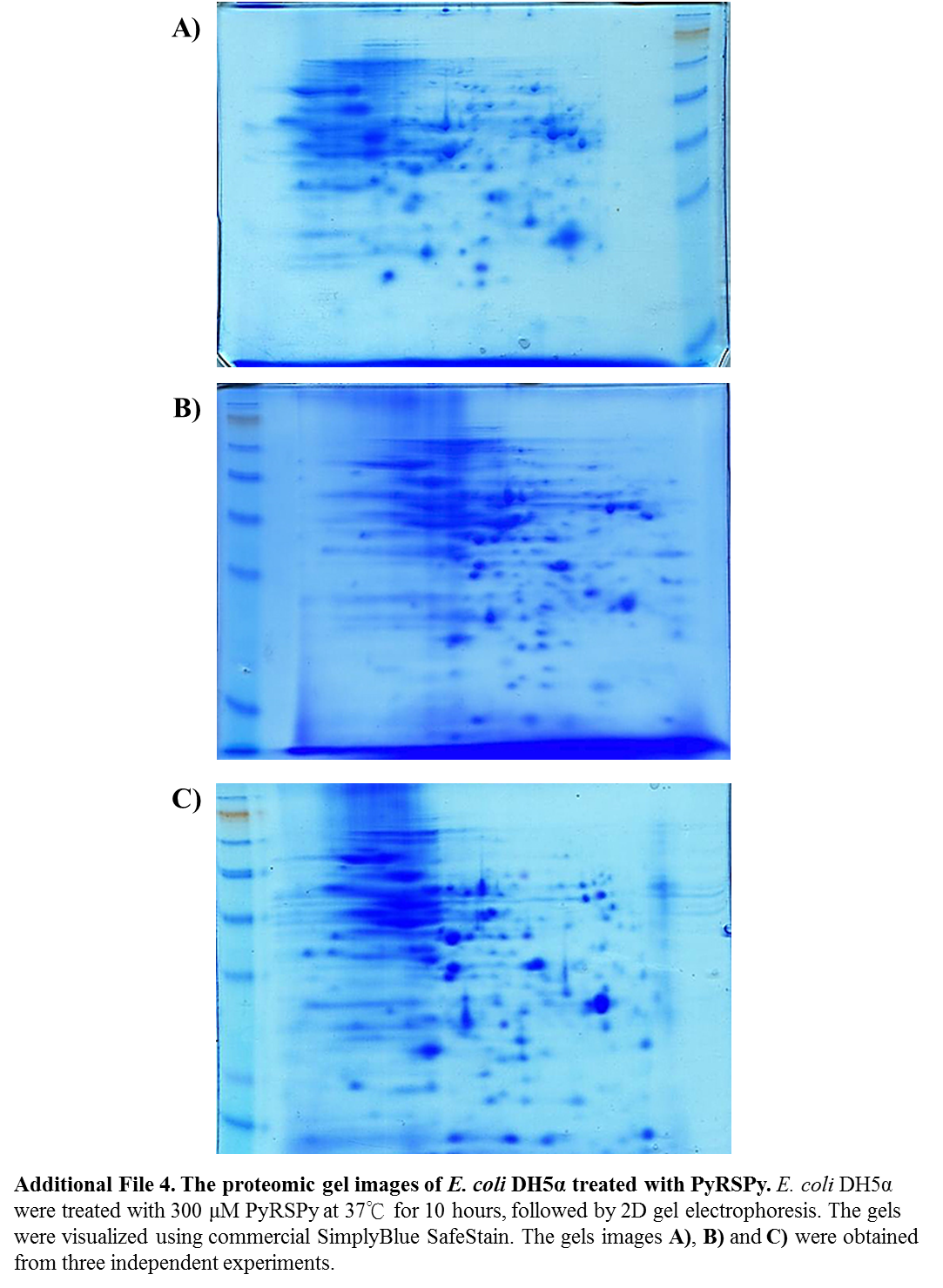

Supplement: Additional file 4 — The proteomic gel images of E. coil DH5α treated with PyRSPy.E. coli DH5α were treated with 300 μM PyRSPy at 37°C for 10 hours, followed by 2D gel electrophoresis. The gels were visualized using commercial SimplyBlue SafeStain. The gels images A), B) and C) were obtained from three independent experiments. [file 1477-5956-11-23-S4.tiff]

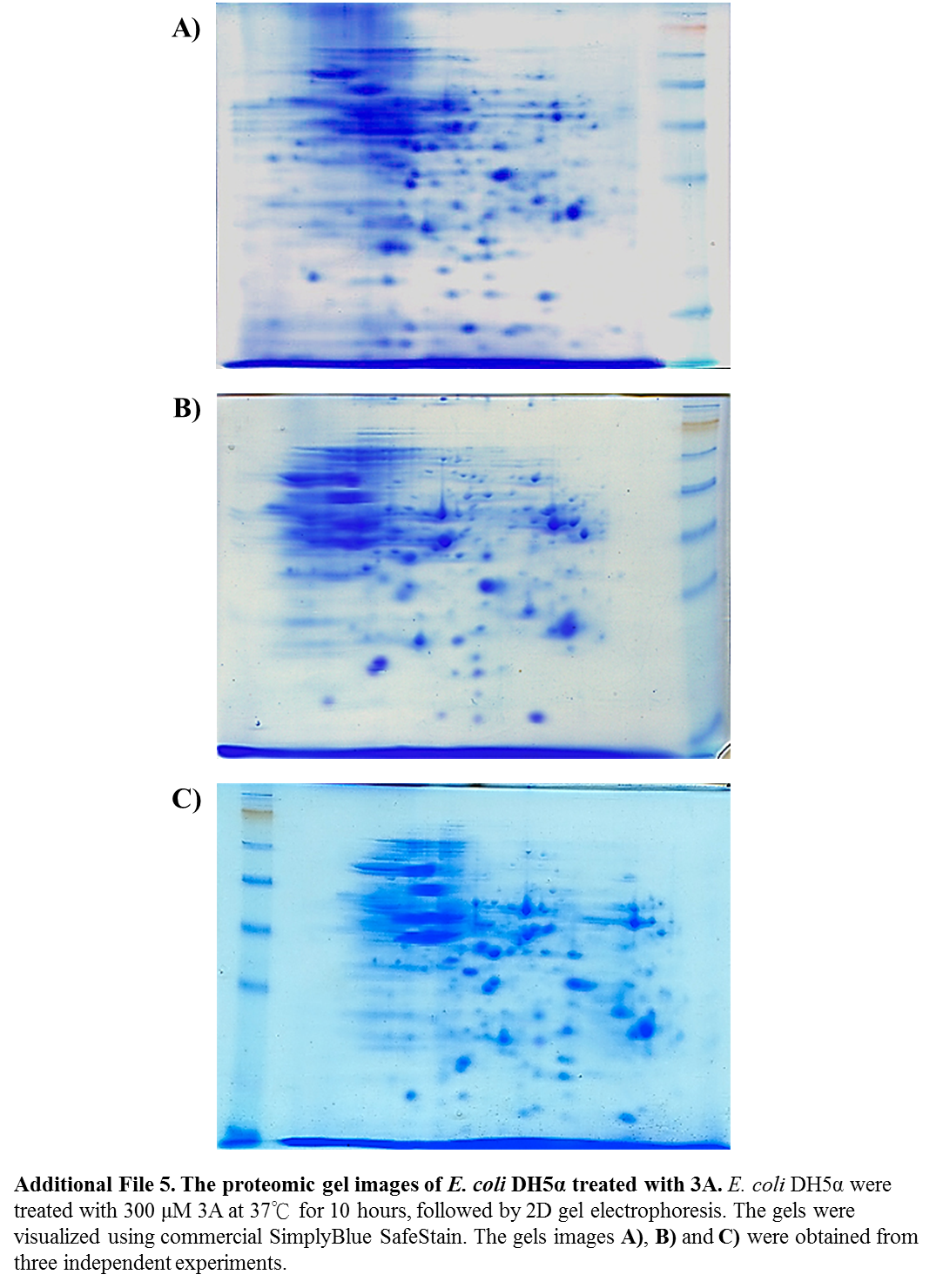

Supplement: Additional file 5 — The proteomic gel images of E. coil D115u treated with 3A. E. coil DH5α were treated with 300 μM 3A at 37°C for 10 hours, followed by 2D gel electrophoresis. The gels were visualized using commercial SimplyBlue SafeStain. The gels images A), B) and C) were obtained from three independent experiments. [file 1477-5956-11-23-S5.tiff]
